# Del Nido *vs.* Blood Cardioplegia: A Comparative Analysis of Postoperative Atrial Fibrillation in Coronary Artery Bypass Grafting Patients

**DOI:** 10.21470/1678-9741-2024-0071

**Published:** 2025-05-23

**Authors:** Hasan Toz, Ali Aycan Kavala, Saygın Türkyılmaz, Yusuf Kuserli, Gülsüm Türkyılmaz, Mehmet Ali Yesiltas, Necdet Kılıçaslan

**Affiliations:** 1 Department of Cardiovascular Surgery, Bakırköy Dr. Sadi Konuk Research and Training Hospital, Istanbul, Turkey; 2 Department of Cardiovascular Surgery, ISTUN Sisli Kolan International Hospital, Istanbul, Turkey

**Keywords:** Cardiopulmonary Bypass, Cardioplegia Solution, Atrial Fibrillation, Del Nido cardioplegia solution, Perfusion.

## Abstract

**Introduction:**

Cardioplegia solution, also called the del Nido solution, has been widely used in pediatric cardiac surgeries, and has recently started to be used in adult cardiac surgeries. In this context, this study aimed to investigate the relationship between the use of del Nido and blood cardioplegia solutions and postoperative atrial fibrillation rates in our clinic.

**Methods:**

The study sample comprised 140 patients who underwent coronary artery bypass grafting. The del Nido and blood cardioplegia solutions were used in 70 (50%) patients. The postoperative atrial fibrillation rates of both groups were compared. Additionally, patients’ preoperative, intraoperative, and postoperative data were evaluated.

**Results:**

The cardiopulmonary bypass duration and defibrillation rate were lower in the del Nido cardioplegia group than in the blood cardioplegia group (P < 0.001). Atrial fibrillation rates on postoperative days one, five, and 30 were significantly lower in the del Nido cardioplegia group than in the blood cardioplegia group (P < 0.001, P < 0.001, and P = 0.007, respectively).

**Conclusion:**

The postoperative atrial fibrillation rate was significantly lower in the del Nido cardioplegia group than in the blood cardioplegia group. In addition, the del Nido cardioplegia solution did not interrupt the surgical flow, thus resulting in less total perfusion, shorter cross-clamping durations, and fewer defibrillation needs. In conclusion, the del Nido cardioplegia solution can be used safely and effectively in coronary artery bypass grafting surgeries.

## INTRODUCTION

**Table:** 

Abbreviations, Acronyms & Symbols				
ACT	= Activated coagulation time		Hct	= Hematocrit
AF	= Atrial fibrillation		HT	= Hypertension
BC	= Blood cardioplegia		ICU	= Intensive care unit
BMI	= Body mass index		IQR	= Interquartile range
CABG	= Coronary artery bypass grafting		LOS	= Length of stay
CAD	= Coronary artery disease		LVEF	= Left ventricular ejection fraction
COPD	= Chronic obstructive pulmonary disease		NG	= Nasogastric
CPB	= Cardiopulmonary bypass		NYHA	= New York Heart Association
CRF	= Chronic renal failure		POAF	= Postoperative atrial fibrillation
DM	= Diabetes mellitus		SD	= Standard deviation
DNC	= Del Nido cardioplegia		VF	= Ventricular fibrillation
ECG	= Electrocardiogram			

Cardioplegia solutions are an essential part of the measures taken to protect the myocardium after aortic cross-clamping in open heart surgeries requiring cardiopulmonary bypass (CPB). Ischemia occurs in myocardial tissue during cardioplegia^[1]^. There are various types of cardioplegia solutions, each with different contents. However, there is still no consensus on the optimal treatment for cardioplegia^[2]^. Blood cardioplegia (BC) solution, a type of extracellular cardioplegia solution, inactivates sodium channels and depolarizes the myocardial membrane. The most common cardioplegia technique involves providing extracellular cardioplegia solutions at 15-20-minute intervals since they provide a short arrest time. The del Nido cardioplegia (DNC) solution is a type of extracellular crystalloid cardioplegia consisting of a 1:4 solution of a blood-crystalloid mixture. The DNC solution contains a plasma-soluble base solution and a crystalloid component. Unlike BC, DNC contains mannitol, magnesium, and lidocaine. Mannitol reduces myocardial edema by increasing osmolarity and scavenging free radicals. In contrast, magnesium prevents the accumulation of intracellular calcium ions, and lidocaine blocks sodium channels and prolongs the duration of hyperpolarizing arrest. A single dose of DNC ensures that cardioplegia arrests for > 90 minutes^[3^,^4]^.

Postoperative atrial fibrillation (POAF) is one of the most common rhythm-related complications after isolated coronary artery bypass grafting (CABG). In this context, this study aimed to investigate the effects of DNC solution, which has been increasingly used for myocardial protection in recent years, on atrial fibrillation (AF) in the early postoperative period.

## METHODS

### Population and Sample

The population of this retrospective clinical study consisted of patients with a left ventricular ejection fraction (LVEF) > 30 who underwent isolated CABG between August 2017 and September 2019 in the Department of Cardiovascular Surgery at the Bakırköy Dr. Sadi Konuk Research and Training Hospital (Istanbul, Turkey). The study protocol was approved in advance by the ethics committee of Bakırköy Dr. Sadi Konuk Research and Training Hospital (approval No. 2019-08-05). The research data were obtained from medical records in the hospital’s database. The consent forms required to perform isolated CABG were obtained from the patients. The study inclusion criteria were as follows: patients who had undergone isolated CABG, were older than 50 years, and had normal preoperative sinus rhythm. The study exclusion criteria were as follows: history of AF, had undergone a surgical procedure other than isolated CABG (*e.g.*, aortic aneurysm, ascending aortic replacement, or valve surgery), had undergone emergency surgery, or had undergone cardiac surgery. Patients who developed AF were included in the study as single patients. Patients who responded to medical treatment and developed AF again were not included in the study as additional patients.

Patients’ age, sex, New York Heart Association functional classification information, history of hypertension, presence of diabetes mellitus (DM), ejection fraction, aortic cross-clamping duration, total CPB duration, number of anastomoses, cardioplegia volume, POAF development, inotropic drug need, length of stay (LoS) in the intensive care unit (ICU), mortality data, and hospital discharge times were recorded.

### Surgical Procedure

The patients were taken to the operating room and placed in the supine position. After the anesthesia team prepared the patient for surgery, a catheter was inserted through the right vena jugularis interna. The heat probe was placed in the nasogastric (NG) region. The mediastinum was reached by opening the sternum with a standard incision and median sternotomy. The left internal mammary artery and saphenous vein grafts were prepared based on the number of vessels to be operated on and the number of grafts to be used. The pericardium was opened and suspended. Patients were heparinized (300-400 U/kg) with an activated coagulation time (ACT) [Abbott i-STAT] of > 400 seconds. As per the planned procedure, CPB was achieved with arterial cannulation from the ascending aorta and two-stage venous cannulation from the right atrium auricle. Autotransfusion devices were not used in any of the operations. Prime solutions were prepared in the same manner for patients in both groups. A cardioplegia cannula was placed in the aortic root.

Cardiac arrest was achieved by administering antegrade cardioplegia under cross-clamping to all patients. The patients were divided into two groups: patients (n = 70) who were given BC solution (BC group) and patients (n = 70) who were given DNC solution (DNC group). BC solution was prepared with 30-40 mEq/L KCl, 12 mEq/L MgSO4, 20 ml of 8.4% NaHCO3, and 200 ml of 5% dextrose at a pH between 7.5 and 7.7, mixed with blood at a 1:4 ratio, and given to the patients every 20 minutes during surgery. The DNC solution was prepared by adding 17 cc of 20% mannitol, 14 cc of 15% MgSO4 (3 mEq/L), 13 cc of 8.4% NaHCO3 (140 mEq/L), and 9 cc of 22.5% potassium (5 mEq/L), and 6.5 cc of 2% lidocaine to 1 liter of Isolyte® S solution, mixed with oxidized blood at a 1:4 ratio and given to the patients at a dose of 20 ml/kg. In the event that surgeries were expected to exceed 90 minutes, maintenance DNC was administered at 60 minutes.

Mild hypothermia (30°C-32°C) was applied during the surgery. Roller pumps and membrane oxygenators were used in all surgeries. The open system is generally preferred for bypass surgeries. A balanced electrolyte solution (Isolyte® S) is generally preferred as the primary solution and is prepared from a crystalloid colloid mixture. Patients with kidney disease and diabetes were not given potassium or glucose. Pump flow was maintained between 2.2 and 2.4 L/min/m^2^, and nonpulsatile and mean arterial pressure was maintained at 60-80 mmHg during cross-clamping. During CPB, the hematocrit (Hct) ranged from 21% to 26%. Distal anastomoses were performed under cross-clamping. After the cross-clamp was removed, the patient was warmed. In both groups, proximal anastomoses were performed under a side clamp. Patients who were in sinus rhythm after the cross-clamp was removed and who developed ventricular fibrillation and thus needed defibrillation were included. Norepinephrine is the first-line treatment for patients with systolic blood pressure < 90 mmHg who are weaned from CPB. CPB was terminated under appropriate hemodynamic conditions, and decannulation was performed. Neutralization was achieved with protamine sulfate until ACT returned to normal levels. Intraoperative parameters were used, including aortic cross-clamping duration, CPB duration, cardioplegia volume, number of bypass grafts, intraoperative defibrillation, and inotropic support (norepinephrine, adrenaline/other). Thirty-six French drains were placed in the mediastinum and left thorax. The median sternotomy was closed with four single steel wires (usually no. 5 or no. 6). After the closure of the skin and subcutaneous tissues, the procedure was terminated, and the patients were followed up at the ICU.

### Premedication and Anesthesia Protocol

For anesthesia induction, 0.003 mg/kg midazolam, 5 pg/kg fentanyl, and 0.1 mg/kg vecuronium bromide were administered. As muscle relaxants, 1 mg/kg succinylcholine and 0.1 mg/kg pancuronium were administered. To maintain anesthesia, 3 µg/kg/min fentanyl infusion was used, and isoflurane was administered via inhalation when necessary. During effective muscle relaxation, the patients were ventilated with 100% O₂ using Ambubags and then intubated. A Foley urinary catheter was inserted to monitor urine output during the operation. Afterward, a central venous pressure catheter was inserted through the right vena jugularis interna. The temperature probe was attached to the patients as the NG. Perioperative continuous blood gas monitoring was performed. Transesophageal ultrasound was not used. Electrolyte levels were kept within normal limits by continuous blood gas monitoring to independently investigate the effect of cardioplegia on rhythm.

### Postoperative Intensive Care Unit Management

Following the transfer of the patients to the ICU after the completion of the surgical procedure, mechanical ventilation was administered routinely for two to 12 hours, depending on the characteristics of the patients. After CABG, the patients were ventilated while in the ICU until their hemodynamics stabilized and respiratory functions returned to normal. The patients’ vital parameters (rhythm, blood pressure, saturation) were constantly monitored. Following the transfer of the patients to the ICU, direct chest radiography was performed to rule out pneumothorax and significant atelectasis and to check the locations of the chest tube and endotracheal tube. After ventilation is started, blood gases are evaluated. PaO₂ values of 75 to 100 mmHg, SaO₂ values of > 95 mmHg, PaCO₂ values of 35 to 45 mmHg, pH values of 7.35 to 7.45, Hct values of 38 to 48%, sodium levels of 133 to 146 mmol/L, potassium levels of 3.5 to 5 mmol/L, and ionized calcium levels of 1.1 to 1.3 mmol/L were ensured via arterial blood gas measurements. If the blood loss exceeded 300 ml and 250 ml in the first and second hours, respectively, or if it exceeded 150 ml thereafter to the point where the hemodynamics were disrupted, the patient would receive a blood transfusion. In cases where the Hct value was < 25% in blood gas analyses, the patient was first given erythrocyte suspension replacement, fresh frozen plasma, whole blood, and platelet replacements when necessary.

### Definition and Treatment of Postoperative Atrial Fibrillation

All patients were followed up at the ICU to continuously monitor their heart rhythm and invasive blood pressure. The diagnosis of AF was made when the 12-lead electrocardiogram (ECG) revealed irregular QRS complexes along with fibrillatory “P” waves of varying sizes, shapes, and durations. AF attacks lasting > 5 minutes were considered POAF. The diagnosis of POAF was made by routine monitoring in the hospital and by ECG during routine follow-ups or check-ups after the 30th day. For AF therapy, low-molecular-weight heparin and electrolytes were administered to each patient who developed POAF as a standard, along with two 10 ml ampoules of 15% magnesium sulfate, which were administered in the form of infusions 20 minutes apart. Afterward, 150 mg/10 minutes amiodarone was administered as the loading dose. Amiodarone was continued as a 1 mg/min infusion during the next six hours, and 0.5 mg/minutes infusions were given during the subsequent 18 hours. After intravenous administration of 1000-1200 mg amiodarone over a period of 24 hours, 3 × 200 mg/day amiodarone was administered orally for the first ten days, 2 × 200 mg/day for the subsequent ten days, and 1 × 200 mg/day for the last ten days^[5]^. We wanted to follow up the POAF rate on the first, fifth, and 30^th^ postoperative days, as in most studies^[5]^.

### Statistical Analysis

The Number Cruncher Statistical System (NCSS, Kaysville, Utah, United States of America, 2007) software package was used for statistical analyses. Descriptive statistics obtained from the research data are expressed as the means and standard deviations, medians and interquartile ranges, frequencies, and percentages. The normal distribution characteristics of the quantitative data were analyzed via the Shapiro-Wilk test and graphs. Independent samples *t*-tests were used for comparisons between two groups featuring quantitative variables determined to conform to a normal distribution. The Mann-Whitney U test was used to evaluate the ICU intubation period (hours), which was not normally distributed. A paired sample *t*-test was used for comparisons of quantitative variables determined to conform to the normal distribution within the group. Pearson’s chi-square test, Fisher's exact test, and Fisher-Freeman-Halton exact test were used to compare the qualitative data. *P*-values < 0.05 were considered to indicate statistical significance.

## RESULTS

**Fig. 1:**
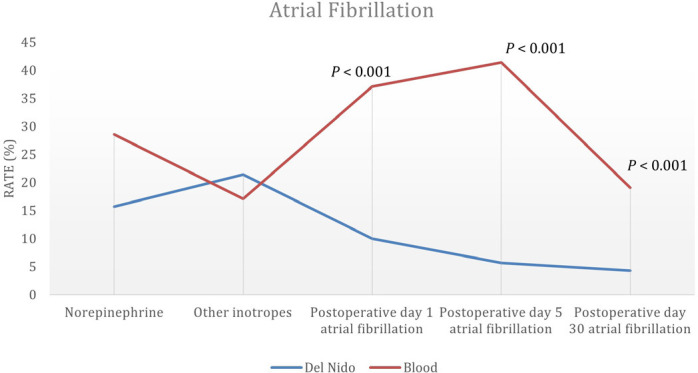
Distribution of patients’ postoperative atrial fibrillation rates on the first, fifth, and 30^th^ postoperative days, and the need for norepinephrine and other inotropes by cardioplegia group.

The mean age of the patients was 66.13 ± 7.7 (min. 52, max. 85) years in BC group and 68.57 ± 7.3 (min. 54, max. 83) years in DNC group. The demographic characteristics of the patients are summarized in Table 1. All patients were in sinus rhythm preoperatively. The number of patients with a history of DM was 35 (50%) in BC group and 35 (50%) in DNC group. Hence, there was no significant difference between the two groups in terms of the number of patients with a history of DM (*P* = 0.999). There were 48 (68.6%) patients with a smoking history in BC group and 38 (54.3%) in DNC group. There was also no significant difference between the two groups in terms of the number of patients with a smoking history (*P* = 0.083). Although the difference was not statistically significant, there was a slight difference between groups. There was no statistically significant difference between the groups in terms of preoperative demographic characteristics (*P* > 0.05). The mean CPB duration was 106.59 ± 11.36 (min. 88, max. 135) minutes in DNC group and 115.33 ± 13.46 (min. 90, max. 150) minutes in BC group, indicating a significant difference between the groups (*P* < 0.001) (Table 2). The mean duration of aortic cross-clamping was significantly shorter in DNC group than in BC group (*P* < 0.001). There was no statistically significant difference between the groups in terms of the number of bypass grafts performed (*P* > 0.05). Defibrillation was applied to nine (12.9%) and 52 (74.3%) patients in DNC group and BC group, respectively, indicating a significant difference between the groups (*P* < 0.001). There was no statistically significant difference between the groups for the mean intraoperative hemoglobin values (*P* > 0.05). Also, there was no statistically significant difference between the groups regarding the use of norepinephrine or other inotropes (*P* = 0.067). Although the difference was not statistically significant, there was a trend towards a difference between groups. The percentages of patients diagnosed with AF on the first, fifth, and 30^th^ postoperative days were significantly lower in DNC group than in BC group (*P* < 0.001, *P* < 0.001, and *P* = 0.007, respectively) (Figure 1). The ICU intubation time significantly increased with increasing blood group (*P* < 0.01). On the other hand, the mean LoS in the ICU was significantly lower in DNC group than in BC group (*P* = 0.009) (Table 3).

**Table 1:** Distribution of patients’ demographic characteristics by cardioplegia group.

		Del Nido (n=70)	Blood (n=70)	*P*-value
Age, mean ± SD		68.57 ± 7.3	66.13 ± 7.7	^a^0.056
BMI, mean ± SD		27.06 ± 1.97	27.3 ± 2.04	^a^0.475
LVEF, mean ± SD		49.67 ± 6.15	48.09 ± 7.62	^a^0.178
Sex	Female	32 (45.7)	35 (50)	^b^0.612
	Male	38 (54.3)	35 (50)	
NYHA functional status	Class 1	4 (5.7)	4 (5.7)	^c^0.979
	Class 2	23 (32.9)	20 (28.6)	
	Class 3	38 (54.3)	41 (58.6)	
	Class 4	5 (7.1)	5 (7.1)	
HT		48 (68.6)	43 (61.4)	^b^0.376
Dyslipidemia		35 (50)	35 (50)	^b^0.999
Sinus rhythm		70 (100)	70 (100)	-
Atrial thrombus		3 (4.3)	6 (8.6)	^d^0.493
CAD		70 (100)	70 (100)	-
Tobacco use		38 (54.3)	48 (68.6)	^b^0.083
Alcohol consumption		8 (11.4)	9 (12.9)	^b^0.796
DM		35 (50)	35 (50)	^b^0.999
COPD		18 (25.7)	15 (21.4)	^b^0.550
CRF		7 (10)	9 (12.9)	^b^0.595
Preoperative stroke		4 (5.7)	6 (8.6)	^b^0.512

a Independent samples *t*-test;

b Pearson chi-square test;

c Fisher-Freeman-Halton exact test;

d Fisher’s exact test

BMI=body mass index; CAD=coronary artery disease; COPD=chronic obstructive pulmonary disease; CRF=chronic renal failure; DM=diabetes mellitus; HT=hypertension; LVEF=left ventricular ejection fraction; NYHA=New York Heart Association; SD=standard deviation

**Table 2:** Comparison of preoperative data between the del Nido and blood cardioplegia groups.

		Del Nido (n = 70)	Blood (n = 70)	*P*-value
Cardioplegia volume, mean ± SD		1208.71 ± 164.53	1071.14 ± 158.52	^a^< 0.001^*^
Cardiopulmonary bypass time, mean ± SD		106.59 ± 11.36	115.33 ± 13.46	^a^< 0.001^*^
Aortic cross-clamping time, mean ± SD		87.61 ± 10.91	96.89 ± 12.83	^a^< 0.001^*^
No. of bypass graft	1	0 (0)	0 (0)	^b^0.322
	2	18 (25.7)	23 (32.9)	
	3	45 (64.3)	34 (48.6)	
	4	6 (8.6)	10 (14.3)	
	5	1 (1.4)	2 (2.9)	
	6	0 (0)	1 (1.4)	
Defibrillation		9 (12.9)	52 (74.3)	^c^< 0.001^*^

a Independent samples *t*-test;

b Fisher-Freeman-Halton exact test;

c Pearson chi-square test

* *P* < 0.01"

SD=standard deviation

**Table 3:** Distribution of patients’ duration of intubation and length of stay in the intensive care unit (ICU) by cardioplegia group.

	Del Nido (n = 70)	Blood (n = 70)	*P*-value
ICU intubation period (hour), median (IQR)	5 (1.5)	6 (3.6)	^a^0.001^*^
ICU stay (day), mean ± SD	2.09 ± 1.05	2.71 ± 1.67	^b^0.009^*^

a Mann-Whitney U test;

b Independent samples *t*-test

* *P* < 0.01

IQR=interquartile range; SD=standard deviation

## DISCUSSION

The findings of this study emphasize the importance of myocardial protection in the context of heart surgery. There are various types of cardioplegia solutions, each with different contents. However, there is still no consensus on the optimal cardioplegia solution^[2]^. To this end, the safety and efficacy of DNC and BC solutions and POAF rates were compared in the 140 patients included in this study. Consequently, POAF and intraoperative defibrillation rates and CPB and aortic cross-clamping durations were significantly lower in the DNC group than in the BC group.

Electrolytes in DNC act through different mechanisms. Mannitol removes free oxygen radicals from the environment by reducing myocardial edema. Magnesium sulfate is a calcium channel blocker that strengthens myocardial contractions and competes with calcium. Sodium bicarbonate has high buffering power. It is prepared by adding potassium chloride, which causes diastolic arrest, and a sodium channel blocker, such as lidocaine, which helps stabilize the myositis membrane by preventing intracellular calcium accumulation.

Lidocaine is classified as a sodium channel blocker and is a frequently used antiarrhythmic agent. Sodium channel blockade increases the refractory process of cardiac myocytes^[6]^. When cardioplegia is treated in an ideal environment without flushing, this action is prolonged because lidocaine remains at a sufficient concentration to continuously affect the myocardium. Additionally, sodium channel blockade helps prevent the adverse effects of hyperkalemic depolarized arrest by polarizing the cell membrane to some extent and preventing the accumulation of sodium and calcium within the cell^[7]^. Lidocaine also emphasizes the importance of achieving diastolic arrest and preventing calcium overload during prolonged ischemia and reperfusion through its action on sodium calcium channels^[8]^. Blocking sodium channel kinetics with lidocaine prevents its influx into cardiomyocytes, thus eliminating the possibility of spontaneous myocardial contraction during ischemic arrest. This reduces the release of troponin I, a marker of myocardial damage, thus providing superior myocardial protection, especially in older hearts^[9]^.

Salinas et al.^[10]^ compared 134 patients who were administered DNC solution with 230 patients who were administered BC solution, and no significant difference was found between the groups about the intraoperative and postoperative characteristics, except for the mean cardioplegia volume, mean cardioplegia dose, or percentage of patients who developed defibrillation after removal of the cross-clamps. In this study, 42% (n = 105) and 8% (n = 13) of patients in BC group and DNC group, respectively (*P* < 0.0001), developed defibrillation after removal of the cross-clamps, indicating that the use of DNC decreased the need for defibrillation in patients who underwent CABG. Similarly, Timek et al.^[11]^ reported that DNC exhibited comparable efficacy in 100 patients who had previously undergone CABG and had lower glucose levels than those who had previously received BC. On the other hand, no significant difference was found between the groups in the incidence of POAF, stroke, reoperation for bleeding, or prolonged intubation either before or after pairing. In contrast, in this study, the POAF rates were significantly lower in DNC group than in BC group.

Shah et al.^[12]^ compared the postoperative parameters of 100 patients who were administered DNC and BC and reported that 40% of the patients in the BC group required defibrillation, whereas only 20% of the patients in the DNC group required defibrillation. The DNC group had a better postoperative arrhythmia profile than the BC group. The rates of POAF, cross-clamping, and CPB durations were significantly lower in the DNC group than in the BC group. Therefore, they concluded that, as in this study, DNC provided better myocardial protection.

In the retrospective study conducted by Alexander Schutz et al.^[13]^, which included 863 patients who underwent CABG, no significant difference was found between 420 patients who were administered DNC and 443 patients who were administered BC regarding preoperative risk variables and outcomes, *i.e.*, mean CPB duration (53.09 minutes *vs.* 52.10 minutes, *P* = 0.206), aortic cross-clamping duration (32.82 minutes *vs.* 33.28 minutes, *P* = 0.967), or operative mortality (2.1% *vs.* 2.5%, *P* = 0.734). However, the use of DNC resulted in significantly lower POAF (23.8% *vs.* 30.7%, *P* = 0.023) and postoperative ventricular tachycardia rates (0.5% *vs.* 3.4%, *P* = 0.002). In general, these results are comparable to the results of this study.

Similarly, in the study conducted by Luo et al.^[14]^, 48 and 41 patients who underwent CABG, valve surgery, or both CABG and valve surgery for the first time were administered DNC and BC, respectively, and the percentage of patients who resumed spontaneous rhythm was significantly greater in the DNC group than in the BC group (97.7% *vs.* 81.6%, *P* = 0.023). On the other hand, contrary to the findings of this study, no significant difference was found between the groups regarding the need for defibrillation.

In Sanrı et al.’s study^[15]^, the number of anastomoses was significantly greater, and the number of aortic cross-clamping events was significantly shorter in the DNC group than in the BC group. In contrast, we found no statistically significant difference between the groups for the number of anastomoses (*P* > 0.05). However, the duration of cross-clamping was prolonged in the BC group. In our study, the durations of cross-clamping and aortic cross-clamping were significantly short. BC was induced every 20 minutes. Therefore, both the cross-clamping and aortic cross-clamping durations were prolonged in the DNC group. Additionally, in a retrospective study conducted by Sanrı et al.^[15]^ involving 255 patients who had undergone isolated CABG, patients who were administered DNC (n = 132) or BC (n = 123) were compared in terms of POAF. Consequently, aortic cross-clamping duration, cardioplegia volume, LoS in the hospital, and POAF risk were found to be lower in the DNC group than in the BC group, in line with this study’s findings. On the other hand, Timek et al.^[16]^ reported that the incidence of AF was significantly greater in the DNC group than in the BC group (*P* < 0.1).

Regarding analyses of CABG patients who underwent high-risk surgery, Yerebakan et al.^[17]^ and Krzysztof Sanetra et al.^[18]^ studied a group of patients who had post-myocardial infarctions. However, the authors did not observe any differences in transfusion rates, LoS, postoperative inotropic support, or 30-day mortality, which is similar to our results.

### Limitations

Our study had several limitations. Its primary limitations were the retrospective and single-center design, as well as the relatively small sample size. Another limitation was the exclusion of patients who underwent emergency operations or reoperations. Large-scale studies, including high-risk patients, are needed to determine whether DNC provides adequate myocardial protection. The fact that troponin and CK-MB values were not routinely checked in all patients constituted an additional limitation of the study. Prospective studies are needed to predict the effects of cardioplegia on rhythm. Any rhythm problems in the patient were detected by instant ECG and monitoring. No rhythm problems were monitored with any Holter monitor. In addition, we compared the POAF rates on the first, fifth, and 30th postoperative days and found that the highest incidence of POAF might have been missed.

## CONCLUSION

DNC solution, previously used in congenital cardiac surgeries, has recently been used in adult cardiac surgeries in the clinic where this study was conducted. The long repetitive dose interval of DNC provides for its use as a single dose, preventing the interruption of surgical flow and shortening the cross-clamping duration and the total perfusion time, thus leading to significantly lower POAF rates. In addition, DNC leads to significantly lower defibrillation rates after cross-clamping than BC. The findings of this study support the relevant findings of studies available in the literature. In conclusion, DNC solution can be used safely and effectively in routine CABG surgeries with lower defibrillation needs and POAF rates.
